# Growth-Promoting Effects of *Pseudomonas glycinae* Strain XJ-33 on Maize Seedlings Under Salt Stress and Its Physiological Responses

**DOI:** 10.3390/plants15142166

**Published:** 2026-07-14

**Authors:** Mengyuan Wen, Xiu Zhang, Guoping Yang, Xuexian Zhang, Haorong Li, Junyan Ma, Ruixin Zhang, Xiquan Li, Liming Lu, Lankun Long

**Affiliations:** 1 Ningxia Key Laboratory for the Development and Application of Microbial Resources in Extreme Environments, College of Biological Science and Engineering, North Minzu University, Yinchuan 750021, China; 19995519852@163.com (M.W.); yang_guoping@126.com (G.Y.); 13258115509@163.com (H.L.); m13258115509@163.com (J.M.); 15706090246@163.com (R.Z.); lxq332022@163.com (X.L.); liminglu0468@163.com (L.L.); 13310291261@163.com (L.L.); 2 Northeast Institute of Geography and Agroecology, Chinese Academy of Sciences, Changchun 130102, China; zhangxx@iga.ac.cn

**Keywords:** salt stress, *Pseudomonas glycinae*, maize, growth promotion

## Abstract

To investigate the regulatory effects of salt-tolerant plant growth-promoting rhizobacteria (PGPR) on crop growth under salt stress and to identify superior bacterial resources for saline–alkaline soil improvement, the maize variety Ningdan 33 was used as the experimental material. Strain XJ-33, a salt-tolerant PGPR isolated from saline–alkaline soil in Ningxia, was selected for inoculation. Based on morphological observation, physiological and biochemical tests, and 16S rRNA gene sequencing, the strain was identified as *Pseudomonas glycinae*. This strain can tolerate extreme conditions of up to 10% NaCl and a pH of 11.0, and exhibits multiple plant growth-promoting traits, including the production of siderophores and indole-3-acetic acid (IAA), as well as ACC deaminase activity. The results showed that inoculation with XJ-33 significantly promoted the growth of maize seedlings under salt stress. Compared with the control, inoculated plants exhibited significant increases in plant height, root length, and biomass (both fresh and dry weights), with the most pronounced increments observed in shoot and root dry weights, which increased by 82.61% and 81.63%, respectively. Physiological and biochemical analyses revealed that leaf SPAD values, chlorophyll content, and nitrogen content increased by 15.00%, 13.18%, and 18.47%, respectively, following inoculation. Additionally, root activity (indicated by dehydrogenase activity) was significantly enhanced. In terms of stress physiology, inoculation improved the osmotic adjustment capacity of the plants; the levels of soluble sugars, soluble proteins, and proline in both leaves and roots increased significantly, whereas the malondialdehyde (MDA) content, an indicator of membrane lipid peroxidation, decreased significantly. Furthermore, the antioxidant enzyme system was positively modulated: superoxide dismutase (SOD) and catalase (CAT) activities were significantly elevated in both leaves and roots, while peroxidase (POD) activity decreased. In conclusion, strain XJ-33 exhibits robust salt tolerance and strong plant growth-promoting capabilities. It can alleviate salt-induced damage in maize by regulating osmotic balance, enhancing antioxidant defenses, and promoting nutrient uptake, thereby demonstrating significant application potential for saline–alkaline soil improvement and the development of microbial agents.

## 1. Introduction

Soil salinization represents one of the most severe challenges confronting global agriculture. According to the Food and Agriculture Organization (FAO), the global area of saline–alkaline land encompasses 1.381 billion hectares (approximately 10.7% of the total land area) [1], with China accounting for approximately 99 million hectares, representing about 10% of its total land area [2]. In recent years, soil salinization in China has intensified, severely restricting the efficient utilization of land resources [3]. Saline–alkaline stress not only deteriorates soil physicochemical properties—such as compaction [4], reduced porosity, and depleted fertility—but also inhibits crop growth and development via ion toxicity and osmotic stress [5], thereby posing a long-term threat to agricultural production and regional ecological security. Consequently, the low-cost and sustainable amelioration and utilization of saline–alkaline land has emerged as a focal point in current agricultural research.

Among various strategies for the amelioration of saline–alkaline land, biological amelioration utilizing PGPR has garnered considerable attention due to its environmental friendliness and lasting efficacy [6,7,8]. The beneficial roles of these bacteria primarily manifest in two ways: first, enhancing plant salt tolerance by regulating soil ion balance and improving the rhizosphere microenvironment [9]; second, providing essential nutrients that can be directly absorbed and utilized by plants [10]. Currently, numerous studies have reported that various PGPR, such as *Bacillus*, *Pseudomonas*, *Azospirillum*, and *Rhizobium* species, can effectively alleviate salt-induced damage in crops [11,12,13,14]. In-depth analyses reveal that the mechanisms by which PGPR mitigate salt stress are multifaceted and synergistic [15]. On one hand, certain PGPR secrete ACC deaminase to reduce excessive ethylene accumulation in plants under salt stress, thereby delaying leaf senescence and growth stagnation [16]. On the other hand, they can trigger induced systemic tolerance (IST) by regulating the antioxidant enzyme system to scavenge excessive reactive oxygen species (ROS) [17], mitigate membrane lipid peroxidation, and assist plants in maintaining intracellular ion homeostasis (e.g., preserving a high K^+^/Na^+^ ratio) [18]. Furthermore, exopolysaccharides (EPS) secreted by some PGPR can chelate harmful soil ions and promote the formation of soil aggregates, physically improving rhizosphere permeability and further attenuating the direct toxicity of salt and alkali to roots [19].

However, although PGPR exhibit significant growth-promoting and stress-alleviating potential under laboratory conditions, exogenously introduced strains often face ecological barriers—such as poor colonization and low survival rates—when confronted with complex saline–alkaline habitats (e.g., high pH, specific ion toxicity, and intense competition from indigenous microbes) [20]. This severely limits their translation from the laboratory to field conditions. Therefore, mining indigenous PGPR resources with natural ecological adaptability from target saline–alkaline regions is considered an effective strategy to overcome these colonization bottlenecks [21].

As a crucial crop for food, feed, and industrial biomass in China, maize (*Zea mays* L.) plays a pivotal role in ensuring national food security [22,23]. However, maize is relatively sensitive to salt stress, particularly during the germination and seedling stages, when salt stress severely inhibits its growth and development, ultimately leading to yield losses [24,25,26]. Therefore, ensuring the normal growth of maize under saline–alkaline conditions remains an urgent practical challenge in agricultural production. Ningxia is one of the regions most severely affected by soil salinization in China, and its extreme saline habitats likely harbor elite indigenous PGPR resources adapted to the local environment. Although *Pseudomonas* is a widely recognized PGPR genus, research on mining highly salt–alkaline-tolerant *Pseudomonas* from saline–alkaline soils in Ningxia, as well as its growth-promoting effects and physiological regulatory mechanisms on local maize cultivars, remains very limited.

Given this, using *Pseudomonas glycinae* XJ-33—isolated from saline–alkaline soil in Ningxia—we hypothesized that inoculation with this strain would multidimensionally and synergistically enhance maize adaptability to salt stress by modulating phytohormone levels, activating the antioxidant scavenging system, and regulating the ion balance network [27,28]. Using the local maize cultivar ‘Ningdan 33’ as the experimental model, this study aims to elucidate the physiological mechanisms by which XJ-33 alleviates salt stress, thereby providing a theoretical basis and elite strain resources for the development of specialized microbial agents for maize and the biological amelioration of saline–alkaline soils.

## 2. Results

### 2.1. Determination of Plant Growth-Promoting Traits and Saline–Alkali Tolerance of the Strain

Assessment of plant growth-promoting traits revealed that although strain XJ-33 lacked the capacities for nitrogen fixation, phosphate solubilization, and potassium solubilization, it exhibited siderophore production, IAA synthesis, and ACC deaminase activity, demonstrating its multifaceted plant growth-promoting potential (Table 1).

**Figure 1:**
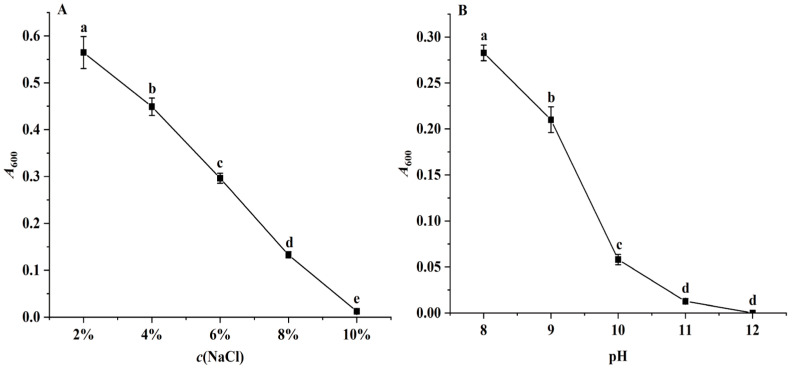
Effects of different NaCl concentrations (**A**) and pH values (**B**) on the growth of strain XJ-33. Note: Data are expressed as means ± *SD* (*n* = 3) of three independent replicates; blank: uninoculated LB medium (zeroed); *p* < 0.05 was considered statistically significant. The different letters in the figure indicate statistically significant differences among the groups (*p* < 0.05).

Uninoculated LB broth (0% NaCl, pH 7.0) was used as the blank for OD_600_ calibration in the salt and alkaline tolerance assays. The results demonstrated that strain XJ-33 grew across a NaCl concentration gradient of 2–10%, with growth peaking at 2% and gradually declining as salinity increased (Figure 1A). Furthermore, the strain tolerated a pH range of 8.0–11.0, whereas growth was completely inhibited at pH 12.0 (Figure 1B).

### 2.2. Physiological and Biochemical Characteristics and Molecular Identification of Strain XJ-33

**Figure 2:**
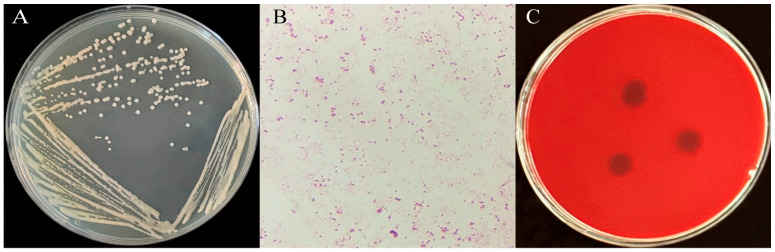
Colony morphology of the strain (**A**), Gram staining ((**B**), 100×), and safety test (**C**).

After 2 days of incubation on LB agar, strain XJ-33 formed circular, convex, opaque colonies (2–4 mm in diameter) with entire margins, smooth and moist surfaces, and creamy-yellow pigmentation (Figure 2A). The cells were Gram-negative short rods (Figure 2B). A safety assessment on Columbia blood agar revealed no hemolytic zones, confirming its non-hemolytic phenotype (Figure 2C).

Physiological and biochemical tests revealed that strain XJ-33 was positive for methyl red, Voges-Proskauer (V-P), catalase activity, and ammonia production. Furthermore, the strain utilized citrate, produced a fluorescent pigment, and hydrolyzed starch and gelatin (Table 2).

**Figure 3:**
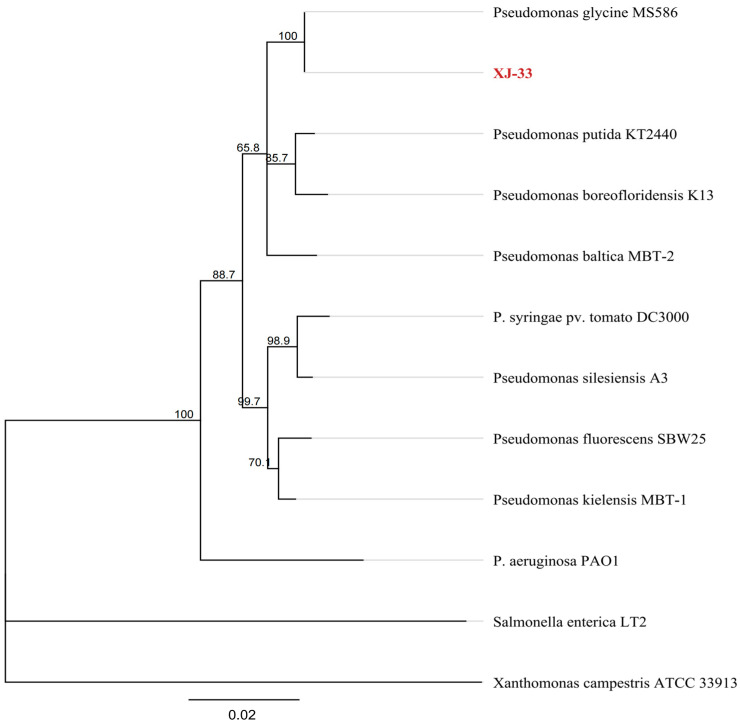
Neighbor-Joining (NJ) phylogenetic tree of strain XJ-33 based on 16S rRNA gene sequences.

The 16S rRNA gene sequence of strain XJ-33 (accession no. PZ437666.1) was compared with the GenBank database using the BLAST algorithm. A neighbor-joining (NJ) phylogenetic tree was constructed using Geneious Prime2025.2.2 with 1000 bootstrap replicates (Figure 3). Integrating morphological, physiological, biochemical, and phylogenetic data, strain XJ-33 was identified as *Pseudomonas glycinae*.

### 2.3. Effects of Inoculation with Strain XJ-33 on Maize Seedling Growth Under Salt Stress

**Figure 4:**
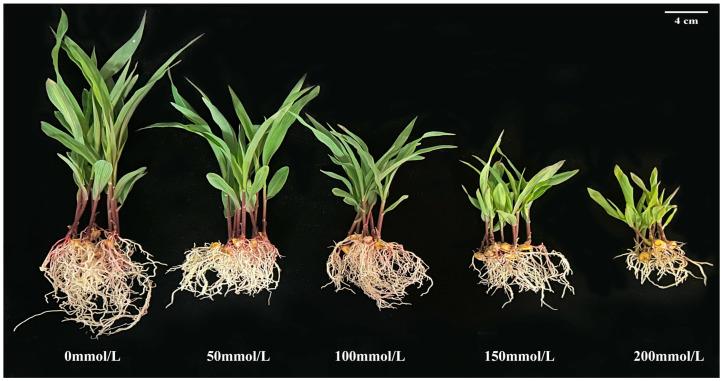
Growth potential of maize seedlings under different NaCl concentrations.

Across a gradient of NaCl concentrations, maize grew normally at 0 and 50 mmol/L but exhibited moderate growth inhibition at 100 mmol/L. However, at 150 and 200 mmol/L, growth was severely stunted, accompanied by substantial plant damage (Table 3, Figure 4). Consequently, 100 mmol/L NaCl was selected as the moderate stress level to evaluate the growth-promoting effects of salt-tolerant PGPR on maize.

**Figure 5:**
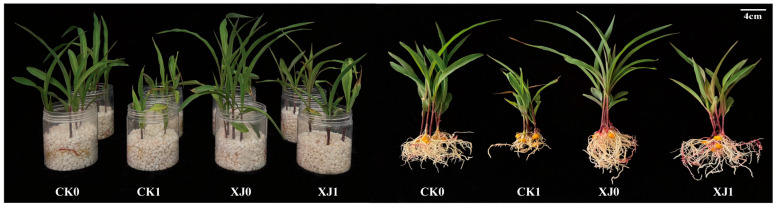
Effects of different treatments on maize seedling growth. CK0, control without salt stress; CK1, control under 100 mmol/L salt stress; XJ0, inoculation group without salt stress; XJ1, inoculation group under salt stress.

**Figure 6:**
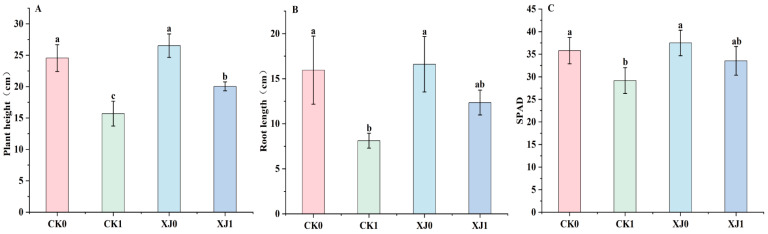
Effects of inoculation with strain XJ-33 on plant height (**A**), root length (**B**), and SPAD (**C**) of maize seedlings under salt stress. CK0, control without salt stress; CK1, control under 100 mmol/L salt stress; XJ0, inoculation group without salt stress; XJ1, inoculation group under salt stress. Error bars represent standard deviation (*n* = 4). Different lowercase letters indicate significant differences among treatments at *p* < 0.05. The same below.

In the hydroponic experiment, inoculation with strain XJ-33 for 14 d promoted maize seedling growth and primary root elongation under 100 mmol/L NaCl conditions, thereby alleviating salt-induced damage (Figure 5). Under normal conditions, compared with the uninoculated control (CK0), the inoculated treatment (XJ0) showed increasing trends in plant height, root length, and SPAD value, with increments of 8.04%, 4.08%, and 4.75%, respectively (Figure 6A–C), whereas stem diameter did not differ significantly. Additionally, XJ0 numerically increased shoot dry weight by 43.18%, although other parameters such as nitrogen content (3.20%), shoot fresh weight (16.13%), root fresh weight (19.95%), and root dry weight (12.50%) did not show statistically significant differences compared with CK0 (Table 4).

Under salt stress, compared with the control (CK1), inoculation (XJ1) significantly increased plant height by 27.71% (Figure 6A). Although root length and SPAD value showed numerical increases of 52.00% and 15.00%, respectively, these differences were not statistically significant compared with CK1 (Figure 6B,C). Stem diameter did not differ significantly between CK1 and XJ1, despite a 6.05% numerical decrease (Table 4). Notably, XJ1 significantly enhanced biomass accumulation; shoot and root dry weights were significantly increased by 82.61% and 81.63%, respectively, and nitrogen content was significantly increased by 18.47%. However, the increases in shoot fresh weight (45.45%) and root fresh weight (40.25%) were not statistically significant compared with CK1 (Table 4).

### 2.4. Impact of Inoculation with Strain XJ-33 on the Physiological Traits of Maize Seedlings Under Salt Stress

**Figure 7:**
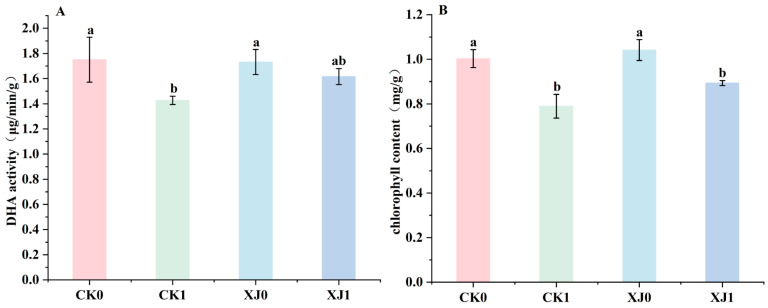
Effects of strain XJ-33 on DHA activity (**A**) and total chlorophyll content (**B**) of maize seedlings under 100 mmol/L NaCl stress. Different letters within the same row indicate significant differences among treatment groups (*p* < 0.05, one-way ANOVA followed by Tukey’s HSD post hoc test).

Root dehydrogenase activity (DHA) and chlorophyll content are pivotal indicators of plant metabolic status. DHA reflects root respiration intensity, whereas chlorophyll is essential for leaf photosynthetic capacity. As shown in Figure 7, salt stress (CK1) significantly reduced DHA and chlorophyll content by 22.67% and 27.06%, respectively, compared with the control (CK0). Under non-stressed conditions, inoculation (XJ0) did not significantly alter either parameter relative to CK0. In contrast, under salt stress, although inoculation (XJ1) resulted in numerical increases of 13.27% and 13.18% in DHA and chlorophyll content, respectively, compared with CK1, these differences were not statistically significant (Figure 7).

**Figure 8:**
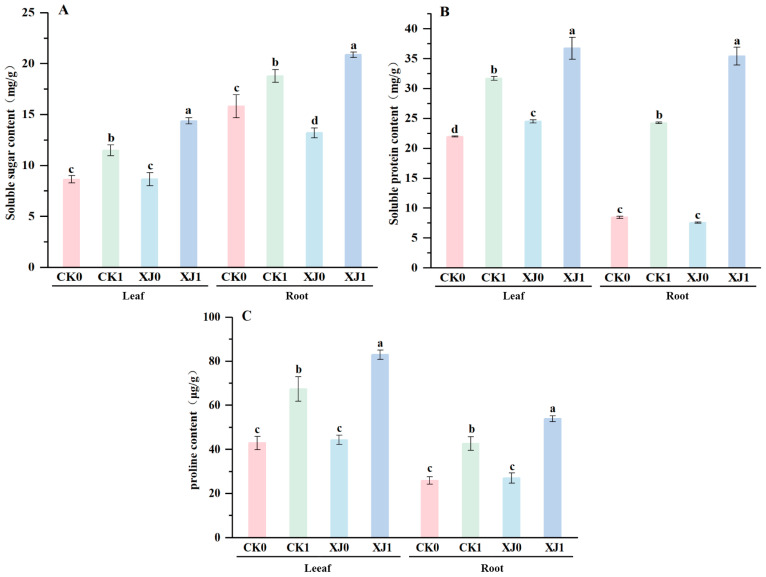
Effects of strain XJ-33 on soluble sugar (**A**), soluble protein (**B**), and proline contents (**C**) of maize seedlings under 100 mmol/L NaCl stress. Different letters within the same row indicate significant differences among treatment groups (*p* < 0.05, one-way ANOVA followed by Tukey’s HSD post hoc test).

Soluble sugars, proteins, and proline function as crucial osmoprotectants, safeguarding cell membrane integrity by reducing cellular osmotic potential and maintaining osmotic balance. As shown in Figure 8, salt stress (CK1) significantly induced the accumulation of these osmolytes compared with the control (CK0). Specifically, the contents of leaf soluble sugar, protein, and proline increased by 32.94%, 44.35%, and 56.96%, respectively, while their root counterparts increased by 18.79%, 186.83%, and 64.76%. Under non-stressed conditions, inoculation (XJ0) numerically increased leaf soluble protein by 11.53% but decreased root soluble sugar and protein by 19.86% and 11.47%, respectively; other parameters remained unchanged. Notably, under salt stress, inoculation (XJ1) significantly enhanced the levels of leaf soluble sugar, protein, and proline by 25.19%, 15.84%, and 23.18%, respectively, as well as their corresponding root levels by 11.08%, 46.01%, and 36.21% relative to CK1. Analysis of tissue-specific accumulation revealed that roots accumulated more soluble sugar, whereas leaves accumulated more soluble protein and proline.

**Figure 9:**
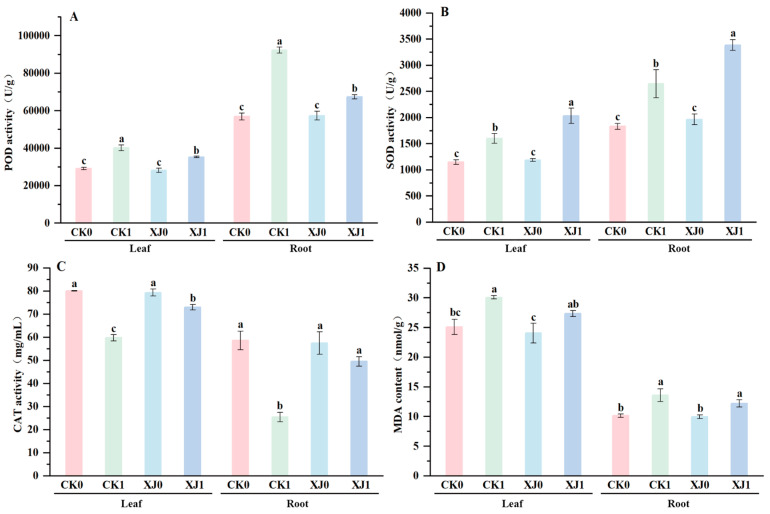
Effects of strain XJ-33 on POD (**A**), SOD (**B**), CAT activities (**C**), and MDA content (**D**) of maize seedlings under 100 mmol/L NaCl stress. Different letters within the same row indicate significant differences among treatment groups (*p* < 0.05, one-way ANOVA followed by Tukey’s HSD post hoc test).

Plants enhance their stress tolerance by upregulating antioxidant enzyme activities under adverse conditions. As shown in Figure 9, salt stress (CK1) significantly elevated leaf POD and SOD activities, as well as MDA content, by 37.77%, 39.31%, and 19.86%, respectively, but significantly reduced CAT activity by 33.99% compared to the control (CK0). In roots, CK1 significantly increased POD and SOD activities and MDA content by 62.32%, 44.37%, and 33.90%, respectively, while significantly decreasing CAT activity by 56.61%. Under non-stressed conditions, inoculation (XJ0) did not significantly alter these parameters relative to CK0. Notably, under salt stress, inoculation (XJ1) differentially regulated these parameters; specifically, in leaves, it significantly decreased POD activity and MDA content by 13.70% and 10.06%, respectively, while significantly enhancing SOD and CAT activities by 27.09% and 22.13%. Similarly, in roots, XJ1 significantly reduced POD activity and MDA content by 37.03% and 11.27%, respectively, while significantly elevating SOD and CAT activities by 27.97% and 94.73%. Tissue-specific comparisons revealed that root POD and SOD activities were higher than those in leaves, whereas leaf CAT activity and MDA content exceeded those in roots.

### 2.5. Principal Component Analysis

**Figure 10:**
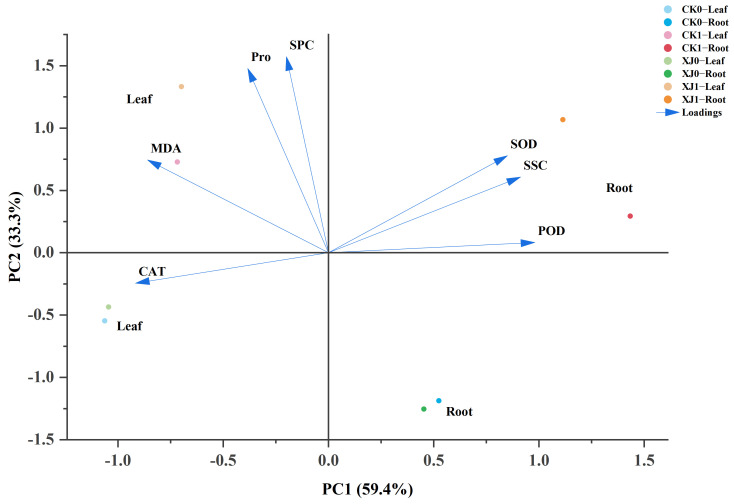
PCA biplot of physiological indicators in maize leaves and roots. PC1 and PC2 explained 59.4% and 33.3% of total variance, respectively. Leaf and root samples exhibited distinct separation along PC1.

The PCA results (Figure 10) revealed that the first two principal components (PC1 and PC2) explained 59.4% and 33.3% of the total variance, respectively, with a cumulative contribution of 92.7%, indicating that they effectively captured the majority of the information in the original dataset. In the score plot, leaf and root samples exhibited distinct spatial separation along the PC1 axis; leaves predominantly occupied the negative region, whereas roots clustered in the positive region. The loading plot further indicated that proline (Pro; PC2 loading = 1.50), superoxide dismutase (SOD; PC2 loading = 0.70), and soluble sugar content (SSC; PC2 loading = 0.55) were closely associated with leaf samples. Additionally, based on PC1 loadings, catalase (CAT; loading = −0.60) was more strongly associated with leaf samples, whereas peroxidase (POD; loading = 0.10) was more closely linked to root samples. This spatial distribution confirms a significant divergence in physiological and metabolic patterns between the aboveground (leaf) and underground (root) compartments of the maize seedlings. Furthermore, different treatments (CK0, CK1, XJ0, XJ1) showed distinct clustering within the principal component space, indicating that both salt stress and inoculation modulated these physiological indicators.

**Figure 11:**
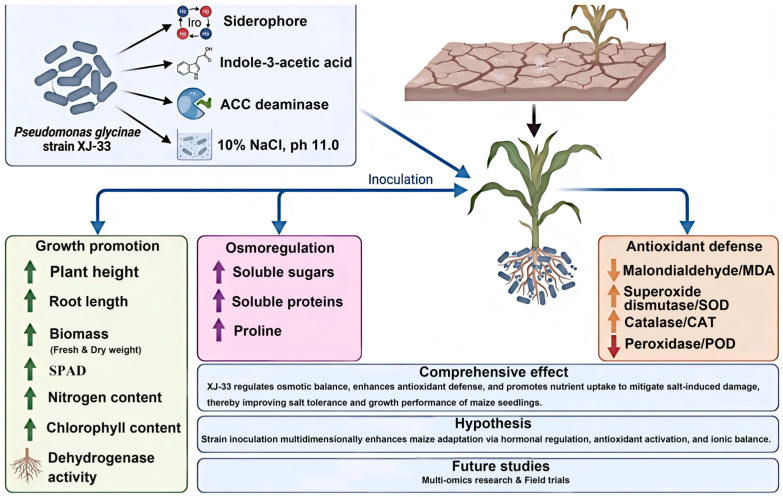
*Pseudomonas glycinae* XJ-33 alleviating salt stress in maize seedlings.

To visually integrate the experimental findings, a schematic diagram illustrating the mechanisms by which *Pseudomonas glycinae* XJ-33 alleviates salt stress in maize seedlings is proposed (Figure 11). As depicted, XJ-33 mitigates salt stress through a multifaceted and synergistic strategy: (1) direct growth promotion and stress adaptation mediated by functional traits (IAA production, siderophore secretion, and ACC deaminase activity); (2) enhancement of “source-sink” synergy by upregulating chlorophyll content and dehydrogenase activity, thereby boosting photosynthetic capacity and respiratory metabolism; (3) induction of osmolyte accumulation (soluble sugars, soluble proteins, and proline) for active osmoregulation; and (4) maintenance of ROS homeostasis, primarily via activation of the SOD-CAT antioxidant system, which reduces membrane lipid peroxidation (MDA) and protects cellular integrity. Collectively, these mechanisms contribute to the enhanced salt tolerance of maize seedlings.

## 3. Discussion

The application of microorganisms to alleviate plant salt stress has garnered increasing attention in recent years. As key microbial resources, PGPR demonstrate unique ecological adaptability under adverse conditions [29]. In this study, a salt-tolerant strain, XJ-33, was isolated from saline soils in the Ningxia region. Its full-length 16S rRNA gene sequence showed 100% identity to *Pseudomonas glycinae* (Figure 3). Furthermore, the physiological and biochemical characteristics of this strain, including Gram staining, citrate utilization, fluorescence, and gelatin hydrolysis (Table 2; Figure 2), were consistent with those reported for *Pseudomonas glycinae* by Jia et al. [30]. Strain XJ-33 exhibited tolerance to 2–10% NaCl and pH 8.0–11.0 (Figure 1). It lacked nitrogen-fixing, phosphate-solubilizing, and potassium-solubilizing capabilities but possessed growth-promoting traits such as siderophore production, IAA secretion, and ACC deaminase activity (Table 1). The strain tested positive for methyl red, V-P, and catalase reactions, could hydrolyze starch, produced ammonia, and displayed no hemolytic activity (Table 2; Figure 2). Although strain XJ-33 was originally isolated from saline–alkali soil, its robust plant growth-promoting traits (e.g., IAA and siderophore production, ACC deaminase activity) and its taxonomic classification within the well-known rhizosphere-colonizing genus *Pseudomonas* suggest that it primarily functions as a rhizosphere bacterium. While direct monitoring of root colonization dynamics (e.g., via GFP-tagging or time-course enumeration) was not conducted, the significant and consistent physiological and phenotypic improvements observed in inoculated seedlings serve as indirect but strong evidence that XJ-33 successfully associated with maize roots and maintained beneficial activity throughout the experiment. Future studies employing fluorescent labeling techniques are warranted to visually and quantitatively elucidate its colonization patterns.

Biomass serves as a crucial indicator for evaluating plant salt tolerance, reflecting carbon assimilation and organic matter accumulation under salt-stressed conditions [31]. Studies have shown that inoculation with salt-tolerant PGPR can promote plant growth through multiple mechanisms, enhancing biomass while mitigating salt-induced damage [32]. The present study found that inoculation with strain XJ-33 significantly increased both the shoot and root biomass (fresh and dry weights) of maize seedlings under salt stress (Table 4). This biomass enhancement is closely associated with morphological and physiological improvements: increased plant height and root length expanded the seedlings’ resource acquisition space, while elevated SPAD values and nitrogen content enhanced photosynthetic capacity and substrate synthesis in leaves. Consequently, strain XJ-33 likely drives dry matter accumulation in maize seedlings by improving nitrogen uptake and photosynthetic performance, thereby bolstering their tolerance to salt stress (Figure 5 and Figure 6).

Leaf chlorophyll content and root DHA are vital physiological indicators for assessing plant salt tolerance [33]. The former reflects photosynthetic assimilation capacity, while the latter represents root respiratory metabolic intensity; notably, these parameters exhibit a significant positive correlation under salt stress [34]. In this study, the total chlorophyll content in maize leaves under salt stress (CK1) was significantly lower than that of the control (CK0). This reduction is likely the combined result of salt stress-induced stomatal closure restricting CO_2_ supply and the disruption of chloroplast structures, which severely impairs light energy absorption and conversion [35,36]. Correspondingly, root DHA activity in the CK1 group also decreased significantly. This synchronous decline in aboveground and underground indicators is not isolated; the sharp decrease in photosynthates under salt stress leads to insufficient carbon supply to the roots, which, coupled with the direct toxicity of salt ions to the mitochondrial dehydrogenase system, collectively triggers a collapse in root metabolic vitality [37]. Upon inoculation with strain XJ-33, both maize chlorophyll content and root DHA activity were significantly elevated (Figure 7). This suggests that strain XJ-33 may not only improve photosynthetic performance by regulating gas exchange to deliver more assimilates to the roots but also directly protect root mitochondrial function to maintain respiratory metabolism. Thus, through a “source-sink” synergistic mechanism, XJ-33 effectively alleviates the dual damage inflicted by salt stress on maize.

Osmoregulation is a core mechanism by which plants resist salt stress, relying on the synergistic accumulation of substances such as soluble sugars, soluble proteins, and proline. Soluble sugars provide energy and stabilize membrane structures, soluble proteins reinforce osmotic potential regulation, and proline plays a pivotal role in lowering osmotic potential and maintaining water balance [38]. This study found that salt stress induced the accumulation of these three osmolytes in maize seedling leaves and roots, representing a passive stress response (Figure 8). However, upon inoculation with strain XJ-33, their contents were further significantly elevated. This phenomenon indicates that XJ-33 may improve the photosynthetic and respiratory metabolism of maize, providing more abundant carbon skeletons and energy substrates for the synthesis of osmolytes, thereby shifting the plant’s strategy from passive defense to active adaptation. Similarly, previous research has confirmed that *Pseudomonas* sp. can significantly increase the content of osmolytes in rapeseed under salt stress [39]. As a strain of the same genus, *Pseudomonas glycinae* XJ-33 demonstrates consistent growth-promoting and stress-resisting potential, namely, enhancing maize salt tolerance by strengthening the osmoregulation system.

The excessive accumulation of reactive oxygen species (ROS) induced by salt stress triggers membrane lipid peroxidation [40], leading to a significant accumulation of MDA, which subsequently exacerbates cell membrane damage and inhibits plant growth. To resist oxidative stress, plants activate the antioxidant enzyme system, in which SOD and CAT constitute the core defense line for ROS scavenging [29,41,42]. This study found that inoculation with XJ-33 significantly reduced MDA content in both roots and leaves of maize seedlings, which is closely related to the significant elevation of SOD and CAT activities and the efficient scavenging of ROS (Figure 9). Notably, inoculation with XJ-33 resulted in a decrease in POD activity, an anomalous phenomenon that suggests a complex regulatory mechanism. Given that the SOD-CAT pathway has already been significantly activated, the reduction in POD activity might be an adaptive regulation to avoid excessive consumption of the substrate (H_2_O_2_) or to reduce energy expenditure on futile metabolism; alternatively, it may indicate that XJ-33 primarily maintains ROS homeostasis by fortifying the SOD-CAT cascade pathway, thereby reducing reliance on the POD pathway. This result aligns with findings in wheat [43] and okra [44], where POD activity did not synchronously upregulate with SOD and CAT in the salt stress response induced by specific PGPR. Taken together, the efficacy of PGPR in regulating the plant antioxidant system is highly heterogeneous, stemming from the complex interplay between strain-specific growth-promoting mechanisms and host responses. The POD downregulation mechanism mediated by XJ-33 warrants further elucidation.

## 4. Materials and Methods

### 4.1. Plant Materials, Bacterial Isolation, and Characterization of Growth-Promoting and Tolerance Traits

Plant materials and bacterial isolation: The experimental plant used was maize (*Zea mays* L.) cultivar “Ningdan 33”, purchased from Yinchuan Runfeng Seed Industry Co., Ltd. For the isolation of the salt-tolerant plant growth-promoting bacterium *Pseudomonas glycinae* sp. XJ-33, soil samples were collected in 2025 from the surface layer (10–20 cm depth) of typical saline–alkali agricultural fields at the foot of Helan Mountain in Yinchuan City, Ningxia (geographical coordinates: 38°30′ N, 106°0′ E). A total of five samples were collected using the five-point sampling method, with each sample containing approximately 1 kg of soil. The samples were preserved at 4 °C until use for the isolation and screening of salt-tolerant plant growth-promoting bacteria. Bacteria were isolated using the serial dilution and spread-plating method. Briefly, 15 g of a thoroughly mixed soil sample was added to a 250 mL Erlenmeyer flask containing 90 mL of sterile water. The flask was agitated for 30 min and then allowed to stand to settle the particles. Subsequently, 1 mL of the supernatant was subjected to 10-fold serial dilutions (10^−1^ to 10^−5^). Aliquots from each dilution were spread onto Luria–Bertani (LB) agar plates, which were inverted and incubated at 28 °C for 18–24 h. Single colonies were selected based on morphological characteristics (color, size, shape, mucoidity, etc.) and purified using the streak plate method. The purified isolates were preserved at 4 °C. Subsequently, a preliminary screening was conducted to evaluate the plant growth-promoting abilities of the purified isolates on maize seedlings. Based on the comparative assessment of plant height and root length, strain XJ-33 exhibited the most significant growth-promoting effects and was therefore selected as the target strain for further detailed physiological, biochemical, and mechanistic investigations.

Characterization of plant growth-promoting traits: The functional traits of the purified strain XJ-33 were evaluated as follows: nitrogen-fixing ability was assessed using Ashby medium; siderophore production was detected using the chrome azurol S (CAS) assay; phosphate-solubilizing ability was determined using Mengjina organic phosphorus and Pikovskaya (PKO) inorganic phosphorus media; potassium-solubilizing ability was tested using Aleksandrov medium [45,46,47]; ACC deaminase activity was evaluated using ADF medium; and IAA production was quantified using the Salkowski colorimetric method [10].

Evaluation of salinity and alkalinity tolerance: The tolerance of strain XJ-33 to salinity and alkalinity was assessed using liquid culture methods. For the salinity tolerance assay, LB liquid media were prepared with different NaCl concentrations (2%, 4%, 6%, 8%, and 10%, *w*/*v*; initial pH 7.0). For the alkalinity tolerance assay, the pH of the LB liquid media was adjusted to 8, 9, 10, 11, and 12 using 1 mol/L NaOH prior to sterilization. All media were autoclaved at 121 °C for 20 min. A 1% (*v*/*v*) inoculum of the bacterial suspension was added to each medium, and the cultures were incubated at 37 °C with shaking at 150 rpm for 18 h. The optical density at 600 nm (OD_600_) was then measured to determine the NaCl tolerance range and the pH growth range of strain XJ-33 [48,49].

### 4.2. Strain Identification

Morphological observations and physiological/biochemical identification were performed using conventional methods, focusing on characteristics such as colony size and color. To characterize strain XJ-33, various tests were conducted, including Gram staining, methyl red (MR), fluorescent pigment production, Voges-Proskauer (V-P), catalase, starch hydrolysis, gelatin liquefaction, ammonia production, and citrate utilization. The safety of the strain was assessed using the Columbia blood agar plate method [50].

Genomic DNA of the strain was extracted using a bacterial genomic DNA extraction kit. The 16S rRNA gene was amplified using the universal bacterial primers 27F (5′-AGAGTTTGATCMTGGCTCAG-3′) and 1492R (5′-GGTTACCTTGTTACGACTT-3′). The PCR reaction mixture (25 μL total volume) contained the following components: 12.5 μL of 2 × Phanta Max Master Mix, 1 μL of template DNA, 1 μL each of the forward and reverse primers, and 9.5 μL of ddH_2_O. The thermocycling profile was performed as follows: initial denaturation at 95 °C for 5 min; 30 cycles of 94 °C for 30 s, 57 °C for 30 s, and 72 °C for 90 s; and a final extension at 72 °C for 10 min, with a holding temperature of 4 °C.

The PCR products were examined via agarose gel electrophoresis, and the successfully amplified fragments were sent to Sangon Biotech (Shanghai, China) for bidirectional sequencing. The resulting sequences were compared with those in the NCBI database using the BLAST algorithm (https://blast.ncbi.nlm.nih.gov/, 28 May 2026). Based on the selected reference sequences, a phylogenetic tree was constructed using Geneious Prime software.

### 4.3. Determination of Growth Parameters in Maize Seedlings Inoculated with Strain XJ-33 Under Salt Stress

Uncoated maize seeds of uniform size were selected and placed in a sterilized beaker. The seeds were surface-sterilized by soaking in 75% ethanol for 12 s. After discarding the ethanol, the seeds were treated with 10% sodium hypochlorite (NaClO) for 15 min, followed by thorough rinsing 5–6 times with sterile distilled water to remove residual disinfectant. The sterilized seeds were subsequently stored in a refrigerator at 4 °C for 3–5 h. Finally, the seeds were placed on water agar plates and pre-germinated in the dark at a constant temperature of 28 °C for 3 days.

Culture bottles were sterilized with 75% ethanol and filled with 450 g of sterile vermiculite. The experiment consisted of five treatment groups: Hoagland’s nutrient solution (control) and Hoagland’s solutions supplemented with 50, 100, 150, and 200 mmol/L NaCl, respectively. The pre-germinated seeds were sown in the bottles (four seeds per bottle) and covered with approximately 1 cm of vermiculite. Each treatment was performed in triplicate, and 80 mL of the corresponding treatment solution was added to each bottle. The seedlings were then transferred to a growth chamber with environmental parameters set to simulate optimal summer conditions for temperature, humidity, and light. During the cultivation period, 20 mL of sterile water was supplemented every 3–4 days. After 14 days, the growth status was observed to determine the appropriate salt stress concentration for subsequent experiments [51].

For preparation of the bacterial inoculum, strain XJ-33 was inoculated into LB liquid medium and cultivated on a shaker at 150 rpm until visible turbidity was observed (logarithmic growth phase). The culture was then transferred to sterile centrifuge tubes and centrifuged at 10,000 rpm for 10 min. After discarding the supernatant, the resulting biomass was resuspended in sterile water, and the cell concentration was adjusted to 1 × 10^8^ CFU/mL (confirmed by OD_600_ measurement and plate counting). This concentration was chosen based on the standard inoculum density widely used and validated in PGPR–plant interaction studies [50,52].

Strain XJ-33 was inoculated into maize seedlings in a hydroponic experiment. The experiment included four treatment groups: (1) non-saline control (CK0), supplied only with Hoagland’s nutrient solution; (2) saline control (CK1), supplied with Hoagland’s nutrient solution + 100 mmol/L NaCl; (3) strain-inoculated group (XJ0), supplied with Hoagland’s nutrient solution and inoculated with strain XJ-33; and (4) combined saline and strain treatment group (XJ1), supplied with Hoagland’s nutrient solution + 100 mmol/L NaCl and inoculated with strain XJ-33. The pH and electrical conductivity (EC) of the treatment solutions were measured, and the osmotic potentials were estimated using the empirical relationship Ψs (MPa) ≈ −0.036 × EC (dS/m). The Hoagland’s nutrient solution without NaCl (CK0) had a pH of 6.71 and an EC of 1894 µS/cm, corresponding to an osmotic potential of approximately −0.07 MPa, whereas the addition of 100 mmol/L NaCl (CK1) increased the EC to 12,735 µS/cm (osmotic potential of approximately −0.46 MPa) while maintaining a similar pH (6.81). This indicates that the salt stress imposed on the plants was primarily driven by osmotic and ionic stress rather than pH alterations. Seedlings from all treatments were grown in a plant growth chamber for 14 days. At the end of the cultivation period, five seedlings were randomly selected from each group. Plant height and root length were measured using a ruler, and stem diameter was measured with a vernier caliper. The relative chlorophyll content (SPAD) and nitrogen content (NC-PCM) were determined using a handheld chlorophyll meter (Beijing Zhongke Weihe Technology Development Co., Ltd., Beijing, China). Furthermore, fresh and dry weights were recorded using an electronic balance.

### 4.4. Determination of Physiological Indices in Maize Seedlings Inoculated with Strain XJ-33 Under Salt Stress

The inoculation method and maize growth conditions for strain XJ-33 were consistent with those described in Section 4.3. After 14 days of growth, physiological indicators were measured following the steps of the corresponding kits.

The contents of soluble sugar, soluble protein, and proline (PRO) were determined using the anthrone colorimetric method, the bicinchoninic acid (BCA) assay, and the acid ninhydrin colorimetric method, respectively. The chlorophyll content was extracted and measured using the acetone extraction method. The MDA content and dehydrogenase (DHA) activity were determined using the thiobarbituric acid (TBA) method and the triphenyl tetrazolium chloride (TTC) assay, respectively. Additionally, the activities of antioxidant enzymes, including CAT, POD, and SOD, were measured using the ammonium molybdate colorimetric method, the guaiacol method, and the nitroblue tetrazolium (NBT) method, respectively [16,53,54,55].

### 4.5. Statistical Analysis

Data are expressed as the mean ± standard deviation (*SD*). Microsoft Excel was used for data processing, and statistical analyses were performed using SPSS 26.0. One-way analysis of variance (ANOVA) was conducted with the significance level set at *p* < 0.05. Figures were generated using Origin 2026. For phylogenetic analysis, the obtained sequences were compared with related sequences from the NCBI database using Geneious Prime, and a phylogenetic tree was constructed via the neighbor-joining method.

## 5. Conclusions

In this study, *Pseudomonas glycinae* strain XJ-33 was identified, exhibiting excellent saline–alkali tolerance by growing robustly at NaCl concentrations ranging from 2% to 10% and pH levels from 8.0 to 11.0. Furthermore, it possesses multiple plant growth-promoting traits, including the production of siderophores, IAA, and ACC deaminase. Under 100 mmol/L NaCl stress, inoculation with XJ-33 significantly promoted maize seedling growth, accompanied by increased dehydrogenase and chlorophyll contents, elevated proline levels in both leaves and roots, and reduced MDA accumulation. Additionally, XJ-33 inoculation increased SOD and CAT activities, while decreasing POD activity. In conclusion, strain XJ-33 effectively alleviates salt-induced growth inhibition in maize by modulating osmolyte accumulation and antioxidant enzyme activities, thereby significantly enhancing plant salt tolerance. Consequently, it represents a highly promising salt-tolerant PGPR. Future research should integrate multi-omics approaches to elucidate the underlying molecular regulatory networks and conduct field trials to evaluate its practical efficacy in maize production under saline–alkali conditions.

## Figures and Tables

**Table 1:** Determination of plant growth-promoting traits of strain XJ-33.

Test Indicators	Results
Nitrogen fixation	−
Organophosphorus solubilizing	−
Inorganic phosphorus	−
Potassium dissolving	−
Siderophore production	+
ACC deaminase activity	+
IAA synthesis	+

Note: “+” means that the strain has this function, and “−” means that it does not have this function.

**Table 2:** Physiological and biochemical characteristics of the XJ-33 strain.

Test Items	Results
Gram stain	−
Methylic-red test	+
V-P test	+
Contact enzyme	+
Citrate test	+
Fluorescent pigment	+
Hydrolysis of starch	+
Gelatin liquefaction	+
Ammonia production test	+

Note: +: Postive, −: Negative.

**Table 3:** Plant height and root length of maize seedlings under different NaCl concentrations.

NaCl Concentration (mmol/L)	Plant Height (cm)	Root Length (cm)
0	27.32 ± 0.44 a	21.82 ± 1.23 a
50	21.28 ± 0.71 b	17.26 ± 0.85 b
100	14.90 ± 0.43 c	10.78 ± 0.39 c
150	10.82 ± 0.37 d	8.32 ± 0.34 d
200	6.82 ± 0.25 e	5.04 ± 0.21 e

Note: Data represent the mean ± standard deviation of five independent measurements. Different letters indicate significant differences at *p* < 0.05 (Duncan’s test).

**Table 4:** Effects of inoculation with strain XJ-33 on biomass of maize seedlings under salt stress.

Maize Biomass	CK0	CK1	XJ0	XJ1
Stem diameter (mm)	3.63 ± 0.24 a	3.99 ± 0.60 a	3.57 ± 0.12 a	3.76 ± 0.44 a
Nitrogen content	14.08 ± 0.82 a	11.78 ± 0.67 b	14.53 ± 0.91 a	13.95 ± 0.81 a
Fresh weight of shoot (g)	0.85 ± 0.13 ab	0.44 ± 0.06 c	0.99 ± 0.09 a	0.64 ± 0.12 bc
Fresh weight of root (g)	1.07 ± 0.21 ab	0.59 ± 0.05 c	1.28 ± 0.33 a	0.83 ± 0.06 bc
Dry weight of shoot (g)	0.11 ± 0.02 b	0.06 ± 0.01 c	0.16 ± 0.03 a	0.09 ± 0.01 b
Dry weight of root (g)	0.22 ± 0.04 a	0.12 ± 0.03 b	0.25 ± 0.03 a	0.22 ± 0.03 a

Data are presented as mean ± *SD* (*n* = 4). Different letters within the same row indicate significant differences among treatments (one-way ANOVA followed by Tukey’s HSD post hoc test, *p* < 0.05).

## Data Availability

The raw data supporting the conclusions of this article will be made available by the authors on request.
